# Incidence of Melanoma in Catalonia, Spain, Is Rapidly Increasing in the Elderly Population. A Multicentric Cohort Study

**DOI:** 10.3390/jcm9113396

**Published:** 2020-10-23

**Authors:** Sebastian Podlipnik, Cristina Carrera, Aram Boada, Nina Richarz, Joaquim Marcoval, Josep Ramón Ferreres, Domingo Bodet, Rosa María Martí, Sonia Segura, Mireia Sabat, Joan Dalmau, Mònica Quintana, Antoni Azon, Neus Curcó, Manel Formigon, María Rosa Olivella-Garcés, Pedro Zaballos, Joaquim Sola, Loida Galvany, Carola Baliu-Piqué, Marta Alegre, Paola Pasquali, Josep Malvehy, Susana Puig, on behalf of the Network of Melanoma Centres of Catalonia

**Affiliations:** 1 Hospital Clinic de Barcelona, University of Barcelona, 08036 Barcelona, Spain; criscarrer@yahoo.es (C.C.); jmalvehy@gmail.com (J.M.); susipuig@gmail.com (S.P.); 2 Institut d’Investigacions Biomediques August Pi I Sunyer (IDIBAPS), 08036 Barcelona, Spain; 3 Biomedical Research Networking Center on Rare Diseases (CIBERER), ISCIII, 28029 Barcelona, Spain; 4 Hospital Universitari Germans Trias i Pujol, Badalona, Universitat Autònoma de Barcelona, 08193 Barcelona, Spain; aramboada@gmail.com (A.B.); ninaricharz@hotmail.com (N.R.); 5 Hospital Universitari de Bellvitge, L’Hospital de Llobregat, 08907 Barcelona, Spain; jmarcoval@bellvitgehospital.cat (J.M.); joseramonferreresriera@yahoo.es (J.R.F.); 6 Hospital Vall d’Hebron, 08035 Barcelona, Spain; dombodet@hotmail.com; 7 Hospital Universitari Arnau de Vilanova, 25198 Lleida, Spain; marti@medicina.udl.cat; 8 Hospital del Mar, IMIM, 08003 Barcelona, Spain; ssegura@parcdesalutmar.cat; 9 Hospital Parc Taulí, 08208 Sabadell, Spain; msabat@tauli.cat; 10 Hospital Santa Creu i Sant Pau, 08041 Barcelona, Spain; jdalmau@santpau.es; 11 Hospital Universitari Sagrat Cor, 08029 Barcelona, Spain; mquintanacodina@gmail.com; 12 Hospital Sant Joan de Reus, 43204 Reus, Spain; azon3030@gmail.com; 13 Hospital Universitari Mútua de Terrassa, 08221 Terrassa, Spain; ncurco@mutuaterrassa.cat; 14 Consorci Sanitari de Terrassa, 08224 Terrassa, Spain; mformigon@cst.cat; 15 Hospital de Sant Joan Despí Moisès Broggi, 08970 Barcelona, Spain; MariaRosa.OlivellaGarces@sanitatintegral.org; 16 Hospital Sant Pau i Santa Tecla, 43003 Tarragona, Spain; pzaballos@aedv.es; 17 Hospital General de Granollers, 08402 Barcelona, Spain; quimsola@yahoo.com; 18 Hospital Dos de Maig, 08025 Barcelona, Spain; Loida.GalvanyRossell@sanitatintegral.org; 19 Hospital d’Igualada, Consorci Sanitari de l’Anoia, 08700 Igualada, Spain; carola.baliu.pique@gmail.com; 20 Hospital Plató, 08006 Barcelona, Spain; marta.alegre@hospitalplato.com; 21 Pius Hospital de Valls, 43800 Valls, Spain; pasqualipaola@gmail.com

**Keywords:** melanoma, incidence, population-based study, epidemiology, Spain, skin cancer

## Abstract

The incidence of melanoma has been increasing worldwide during recent decades. The objective of the study was to analyse the trends in incidence for in situ and invasive melanoma in the Spanish region of Catalonia during the period of 2008–2017. We designed a cross-sectional study with an age-period-cohort analysis of melanoma patient data from the Network of Melanoma Centres in Catalonia. Our database covered a population of over seven million and included a total of 8626 patients with incident melanoma. The main outcome measures were crude and age-standardised incidence rates to the European 2013 standard population. Joinpoint regression models were used to evaluate the population trends. We observed an increase in the age-standardised incidence rate (per 100,000 population) of all melanoma subtypes from 11.56 in 2008 to 13.78 in 2017 with an average annual percent change (AAPC) of 3.5%. This incidence increase was seen exclusively in the older population. Moreover, the stratified analysis showed a statistically significant increase in the age-standardised incidence rate for invasive (AAPC 2.1%) and in situ melanoma (AAPC 6.5%). In conclusion, the incidence of melanoma has continued to increase in the elderly population over recent decades, with a rapidly increasing trend of in situ melanomas and the lentigo maligna subtype.

## 1. Introduction

Melanoma is responsible for 1%–2% of all cancers worldwide and accounts for only 5% of all malignant skin tumours. However, it is one of the most aggressive forms of cancer and causes 75–90% of skin cancer-related deaths [1]. In addition, melanoma is one of the cancers that presents earlier in life and is one of the solid neoplasms that produces the greatest number of potential life years lost [2,3,4].

Despite the fact that major primary prevention campaigns have been implemented in recent decades [5,6,7,8,9], the number of melanoma cases continues to rise every year [10,11]. Recent data from the Surveillance, Epidemiology, and End Results (SEER) registry indicate that the incidence of melanoma is rapidly increasing, especially in older patients [10]. However, what it is even more worrying is the fact that there has been an increase in the incidence of melanoma in young adults, especially women between 25 and 39 years of age, often with high associated mortality [12].

In addition, incidence and mortality vary widely among different geographical areas, due to diverse ethnicities and social conditions, with the white race being the most affected in raw numbers [4]. WHO estimates of age-standardised incidence rates worldwide vary widely and show higher incidences in Australia and New Zealand with 33.6 cases per 100,000 population/year, followed by Europe with rates between 9 and 18.8 cases per 100,000 population and the United States with 12.6 cases per 100,000 population [11]. Specifically in Spain there is an incidence rate of 6.4 cases per 100,000 inhabitants per year and a prevalence of skin melanoma in the last five years of 18,181 cases [11]. Although the incidence of melanoma has been increasing globally over recent decades, SEER data has shown that for the first time, the mortality rate decreased by 17.9% in the period 2013–2016 [11,13]. These data may suggest that new drugs, introduced during the last decade for the management of metastatic and locally advanced melanoma, appear to improve survival [13,14].

In the present study, we analysed the population-based age-specific data for cutaneous melanoma in the Catalan population. A thorough knowledge of the incidence of melanoma in Spain is necessary to improve public health policies.

## 2. Material and Methods

This is a descriptive study with an age-period-cohort analysis of melanoma in the region of Catalonia, located in north-east Spain. We obtained the data on the Catalonian population from the Statistical Institute of Catalonia (IDESCAT) [15], and totals 16.23% of the Spanish population with 7,496,276 inhabitants in 2017, and corresponds to a predominantly white population. The melanoma incidence data was obtained from the Catalan registry of melanoma (Xarxa-melanoma) for the period of 2008–2017. The Xarxa-melanoma is a collaborative prospective database in which a total of 19 hospitals participate, covering most of the population of Catalonia. Cases diagnosed and monitored exclusively in private centres were not included in the database. These cases are a minority in Catalonia since most melanoma patients are seen in public hospitals, especially in the case of invasive tumours.

The research protocol received approval from the Research Ethics Committee of the Hospital Clinic of Barcelona (IRB number: HCB/2015/0298). All data were collected prospectively during the time period. This study followed the “strengthening the reporting of observational studies in epidemiology” (STROBE) statement [16].

We analysed all cases of invasive and in situ cutaneous melanoma, including all ages. To evaluate age periods, we used equally spaced two-year calendar periods to analyse differences in time. Category variables were compared using Pearson’s x^2^ test, or Fisher’s exact test, when the expected observations were fewer than five. For continuous variables, we used the mean and standard deviation and linear model ANOVA test.

Annual incidence rates were age-standardised to the European standardised population [17] by the direct method to remove the confounding effect of age and to make valid comparisons between the incidence rates from different countries and 95% confidence intervals for age-adjusted rates were calculated using the Fay and Feuer method [18].

To examine the population trends in age-standardised incidence rate according to sex and histological subtype (invasive vs in situ), joinpoint regression models were used. We calculated the average annual percent change (AAPC) for each variable independently using a fixed period between 2008 and 2017 for an easier comparison of the subgroups. Moreover, we calculated the average annual percentage change (APC) with a maximum number of possible joinpoints set to one, based on the number of data points in the series [19]. A permutation test was used to determine the location of the joinpoints, when the change in trend was statistically significant. The resulting slope was recorded as the APC.

Statistical analyses were performed using the computing environment R and RStudio [20,21], and the Joinpoint Regression Software, version 4.8.0.1 (National Cancer Institute) [22]. All statistical analyses were two sided and assessed for statistical significance at *p* < 0.05.

## 3. Results

**Figure 1:**
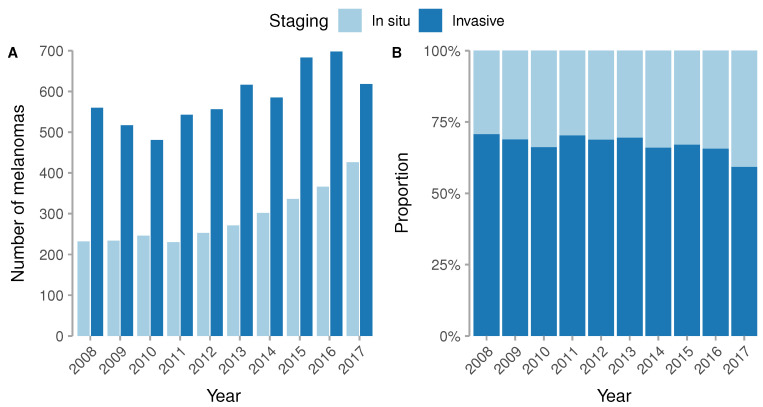
Distribution of invasive and in situ melanomas.

During the study period of 2008 to 2017, we identified a total of 8753 new cases of melanoma in the Catalonian region, of which 2896 were in situ and 5857 invasive. The analysis stratified by two-year groups revealed that there were no differences between the proportion of men and women over that time; however, the age of presentation of melanoma increased from a mean of 58.2 (SD 17.3) years in 2008 to 63.3 (SD 17.1) in 2017 (*p* < 0.001). Table 1 Moreover, there was a clear trend to diagnose a higher proportion of in situ than invasive melanomas, and the proportion of the lentigo maligna subtype also increased from 13.9% to 22.6%. (Table 1 and Figure 1).

**Figure 2:**
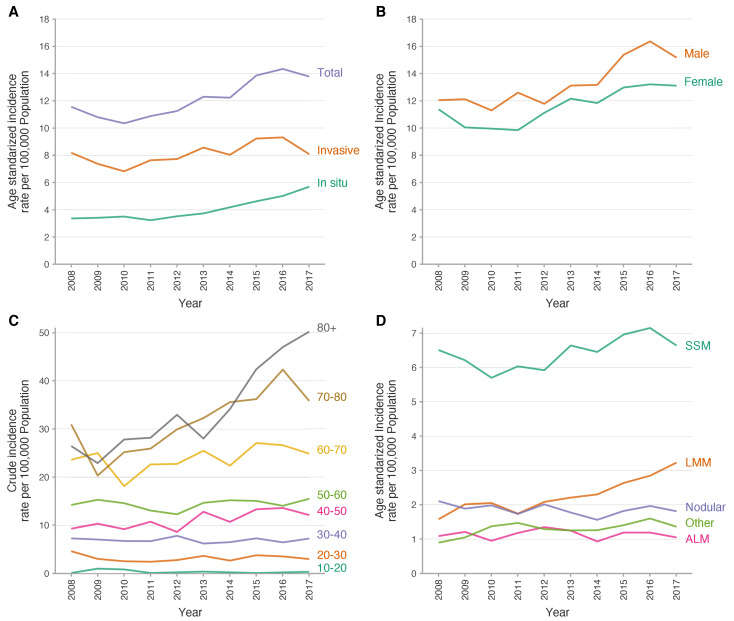
Trends in the incidence of melanoma in Catalunya. Abbreviations: ALM, acral lentiginous melanoma, LMM, lentiginous malignant melanoma; SSM, superficial spreading melanoma.

**Figure 3:**
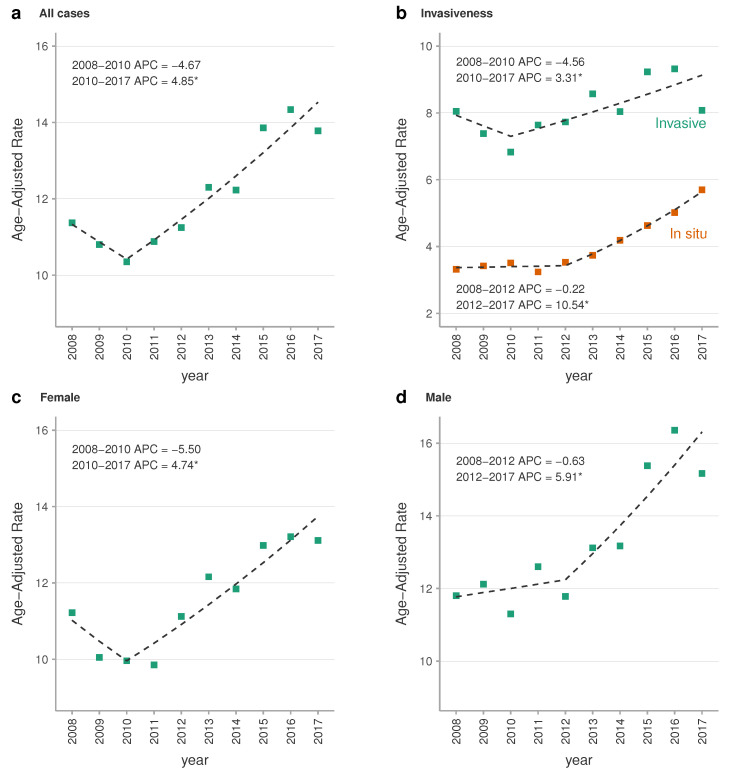
Joinpoint regression analysis of melanoma age-standardised incidence rate. European age-standardised incidence rate trends for melanoma lesions in Catalonia in the period 2008–2017. Squares shapes represent the observed values and dashed lines represent the joinpoint models. (*) APCs were significantly different from zero at alpha = 0.05. Abbreviations: APC, annual percent change.

Age-standardised incidence rate of melanoma data is summarised in Table 2. Between 2008 and 2017, there was a significant increase in the age-standardised incidence rate of melanoma per 100,000 population/year from 11.56 (95% CI, 11.38–11.75) in 2008 to 13.78 (95% CI, 13.57–13.98) in 2017. This means a 19% global increase with an average annual percentage change (AAPC) of 3.5% (95% CI, 2.1%, 5.0%) and an annual percentage change (APC) of 4.9% (95% CI, 2.7%, 7.0%). Moreover, the stratified curves for age-standardised incidence for subtypes of melanoma showed an increased trend for both groups. However the magnitude was greater for in situ melanomas (AAPC of 6.5%; 95% CI, 4.4%, 8.6%) than invasive melanomas (AAPC of 2.1%; 95% CI, 0.1%, 4.1%). The stratified analysis by sex showed a higher age-adjusted incidence of melanoma in men over the whole study period, and also showed a similar upward trend in both groups with an AAPC of 3.9% (95% CI, 2.2%, 5.6%) and 3.2% (95% CI, 1.6%, 4.9%) for men and women respectively (Figure 2 and Figure 3).

**Figure 4:**
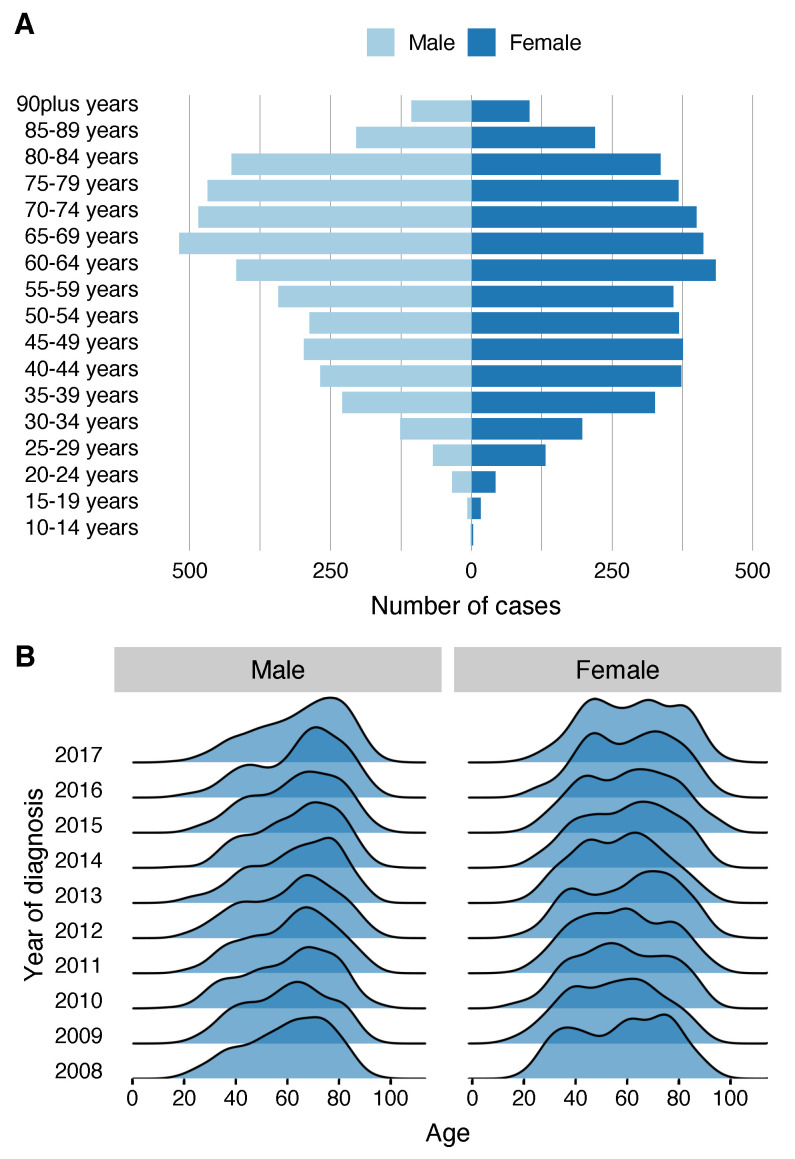
Pyramid plot in (**A**) shows that cases occur at younger ages in women showing a double peak of incidence; however, in men they present in a single peak of incidence at older ages. (**B**) shows a ridgeline with the distribution trend across the years of the study.

Age-specific crude incidence rates by 10-year age bands for 2008 to 2017 showed a stable incidence rate during the follow-up period in patients under 60 years; however, from the 60–70 year age group, a rapid increase in incidence is seen as the age of presentation increases. Additionally, in 2017, the crude incidence rate for patients over the age of 80 years was 50.25 cases per 100,000 population (Figure 2). Moreover, a pyramid plot stratified by sex showed that in women there is a double peak of incidence of melanoma in the 45–49 year bracket and then between 60 and 64 years, while men present a single peak of incidence later in life, between 65 and 69 years (Figure 4A). Moreover, the mean age at presentation of melanoma was 59.1 years in women (SD 17.7) and 62.8 years (16.6) in men (*p* < 0.001). A ridgeline plot shows the distribution of cases represented as density over the years in the study, clearly showing a bimodal distribution of cases in women and only one peak of incidence in men which is maintained during the study period (Figure 4B).

Finally, the analysis of melanoma subtypes showed that the age-standardised incidence rate of superficial spreading melanoma and lentigo maligna melanoma is increasing, while the proportion of patients with nodular, acral lentiginous and other subtypes of melanoma remains constant (Figure 2).

## 4. Discussion

Our study demonstrates that the incidence of melanoma in Catalonia continued to increase during the 2008 to 2017 period with a rising trend which mainly affected elderly patients. This increase has been consistently observed in other countries with predominantly Caucasian populations [23,24,25] and this rising trend is consisted with a previous period studied in Catalonia [26]. Despite this increase in incidence, the age-standardised incidence rate (ASIR) of invasive melanoma in Spain is relatively low (ASIR 6.4) in the GLOBOCAN registry in comparison with other European Mediterranean countries such as France (ASIR 13.6), Italy (ASIR 12.4), Greece (ASIR 8.7) and Malta (ASIR 8.0) [23]. This could be explained in part by an underestimation due to the lack of a comprehensive national Spanish melanoma registry. Another explanation could be the patient’s phenotype (e.g., skin type, red hair) [27] and genotype (e.g., MC1R polymorphisms status), sunlight incidence during the year [28], sun-exposure behavior [29] and the Mediterranean diet which has also been reported as a favorable modifying factor of melanoma incidence [30]. A further possible explanation is the large foreign population living in Catalonia mainly coming from countries with a pigmented skin population and/or low melanoma incidence. In 2017, there were 1,041,362 immigrants which corresponds to 13.9% of the Catalan population: 27.7% were from Africa and 17.4% from South America which could dilute the cases of melanoma and lower the incidence rate [15].

Recent epidemiological data have shown a steady increase in the incidence of melanoma of 3% per year and it is expected that by 2030 the number of melanoma cases will double that of 2016 [25]. Our data showed an AAPC of 3.5% and APC of 4.9% during the last period which is consistent with what has been reported in the literature. If these predictions are accurate, the expected increase in melanoma prevalence will be associated with higher treatment costs, as some of these patients will require new target treatments and immunotherapy [25].

The analysis by melanoma subtype showed that the age-standardised incidence rate of invasive melanomas increased slightly with an AAPC of 2.1% while in situ melanomas almost doubled their incidence with an AAPC of 6.5%. These results are consistent with the findings of a large European study by Sacchetto et al. [31], who observed a significant incidence increase in both invasive (AAPC 4.0%) and more markedly for in situ melanoma (AAPC 7.7%). An increase in incidence of these proportions could mean a lifetime risk of developing in situ melanoma of 1 in 58 people [32]. Although the incidence of invasive melanoma is not as marked as in situ melanoma, the absolute number of thick melanomas continues to rise, which could have an serious impact in the mortality of the patients and costs related to the new treatments [32].

We have observed in this cohort that the proportion of invasive melanomas is higher than in situ melanomas with a ratio of approximately 2:1. This ratio is in line with other European cohorts [31]. On the other hand, in the SEER registry the ratio of invasive versus in-situ melanoma is close to 1:1 [10,33]. One possible explanation is that many low-risk patients are normally seen in private clinics and some of them are not entered in the regional registries.

The increasing trend in incidence during the study decade was observed in the 60–70 age group and above, while the younger age groups remained fairly stable. Moreover, the histological subtype that increased notably during this period is the lentigo maligna subtype. This trend was observed in the study by Swetter et al. who reported a significant increase in melanoma in-situ in the older population. They also found that lentigo maligna accounted for 79–83% of the total diagnoses of the in-situ subtype [34]. All these findings could be explained by accumulated harm from UV (ultraviolet) radiation since childhood as UV exposure and resulting from occupations with high sun exposure (>20 years) leading to the development of melanoma [27]. Large public initiatives to decrease sun exposure have been carried out for many years [5,6,7,8,9,35], but unfortunately, these campaigns will not benefit older people today who could have already had intense sun exposure during childhood. This could explain the significant increase in incidence from age 50 years onwards which we have seen in our study and the important increase in the lentigo maligna melanoma subtype. Another possible explanation for the increase in incidence has partially been attributed to the development of highly sensitive diagnostic techniques during the last decade, principally the use of dermatoscopy which has allowed the detection of clinically unidentifiable tumors [36].

In the stratified analysis by sex, we observed that in both sexes the age-standardised incidence rate showed a rising trend with an AAPC of 3.9% for men and 3.2% in women; moreover, the age-standardised incidence rate was higher in men throughout the study period. Different authors have described that melanoma incidence differs between men and women by age, with women having higher rates of melanoma early in life and men at later stages of life [37,38,39]. One biological explanation for this difference could be related to the hormonal profile. It has been seen that high levels of oestrogen during a woman’s fertile period can promote the formation of reactive oxygen species which seem to favour the development of melanoma seen in younger women [40].

### Strengths and Limitations

A strength of this study was the ability to collect all data prospectively from the main reference hospitals in Catalonia. As a limitation of the study, private centres are not included, resulting in a possible underestimation of a proportion of early melanomas as well as possible differences in melanoma subtypes. Furthermore, this study does not include mortality data.

## Figures and Tables

**Table 1:** Basal characteristics of the tumours.

	2008–2009 (*N* = 1543)	2010–2011 (*N* = 1500)	2012–2013 (*N* = 1696)	2014–2015 (*N* = 1906)	2016–2017 (*N* = 2108)	Total (*N* = 8753)	*p* Value
**Gender**							0.496
Female	789 (51.1%)	751 (50.1%)	897 (52.9%)	971 (50.9%)	1060 (50.3%)	4468 (51.0%)	
Male	754 (48.9%)	749 (49.9%)	799 (47.1%)	935 (49.1%)	1048 (49.7%)	4285 (49.0%)	
**Age**							<0.001
Mean (SD)	58.2 (17.3)	59.6 (17.3)	60.4 (17.3)	61.9 (17.0)	63.3 (17.1)	60.9 (17.3)	
**Staging**							<0.001
In situ	466 (30.2%)	476 (31.7%)	524 (30.9%)	638 (33.5%)	792 (37.6%)	2896 (33.1%)	
invasive	1077 (69.8%)	1024 (68.3%)	1172 (69.1%)	1268 (66.5%)	1316 (62.4%)	5857 (66.9%)	
**Breslow index**							0.219
Mean (SD)	2.2 (6.0)	2.4 (4.5)	2.4 (3.6)	2.2 (4.1)	2.1 (3.2)	2.3 (4.3)	
**Tumour Location**							0.001
Trunk	551 (41.4%)	527 (40.2%)	573 (38.8%)	740 (43.1%)	888 (45.1%)	3279 (42.0%)	
Head and neck	278 (20.9%)	302 (23.1%)	310 (21.0%)	371 (21.6%)	412 (20.9%)	1673 (21.4%)	
Lower limbs	254 (19.1%)	231 (17.6%)	280 (18.9%)	268 (15.6%)	298 (15.1%)	1331 (17.1%)	
Upper limbs	145 (10.9%)	148 (11.3%)	190 (12.9%)	219 (12.8%)	237 (12.0%)	939 (12.0%)	
Acral	97 (7.3%)	85 (6.5%)	107 (7.2%)	92 (5.4%)	107 (5.4%)	488 (6.3%)	
Mucosa	7 (0.5%)	17 (1.3%)	18 (1.2%)	27 (1.6%)	27 (1.4%)	96 (1.2%)	
Missing values	211	190	218	189	139	947	
**Histological subtype**							<0.001
SSM	871 (63.9%)	806 (61.7%)	884 (60.2%)	965 (59.5%)	1011 (55.1%)	4537 (59.7%)	
LMM	190 (13.9%)	183 (14.0%)	227 (15.5%)	306 (18.9%)	414 (22.6%)	1320 (17.4%)	
Nodular	180 (13.2%)	176 (13.5%)	193 (13.1%)	180 (11.1%)	189 (10.3%)	918 (12.1%)	
Acral lentiginous	65 (4.8%)	51 (3.9%)	85 (5.8%)	73 (4.5%)	74 (4.0%)	348 (4.6%)	
Mucosal	4 (0.3%)	13 (1.0%)	11 (0.7%)	16 (1.0%)	20 (1.1%)	64 (0.8%)	
Desmoplastic	10 (0.7%)	14 (1.1%)	10 (0.7%)	8 (0.5%)	15 (0.8%)	57 (0.8%)	
Spitzoid	3 (0.2%)	1 (0.1%)	8 (0.5%)	11 (0.7%)	21 (1.1%)	44 (0.6%)	
Nevoid	3 (0.2%)	1 (0.1%)	1 (0.1%)	4 (0.2%)	8 (0.4%)	17 (0.2%)	
Other	37 (2.7%)	62 (4.7%)	50 (3.4%)	60 (3.7%)	82 (4.5%)	291 (3.8%)	
Missing values	180	193	227	283	274	1157	

Only includes the evaluation of invasive melanomas.

**Table 2:** Incidence of melanoma in Catalonia.

Year	Population	Cases	Crude Rate	ASIR	95% CI
**Melanoma (Including In Situ Melanoma)**					
2008	7,298,313	792	10.85	11.56	[11.38–11.75]
2009	7,416,605	751	10.13	10.80	[10.62–10.97]
2010	7,462,044	727	9.74	10.35	[10.18–10.52]
2011	7,501,853	773	10.30	10.88	[10.7–11.06]
2012	7,515,398	809	10.76	11.25	[11.07–11.43]
2013	7,478,968	887	11.86	12.30	[12.11–12.49]
2014	7,433,894	887	11.93	12.23	[12.05–12.43]
2015	7,424,754	1019	13.72	13.86	[13.65–14.06]
2016	7,448,332	1064	14.29	14.34	[14.14–14.55]
**2017**	7,496,276	1044	13.93	13.78	[13.57–13.98]
**Invasive melanoma**					
2008	7,298,313	560	7.67	8.19	[8.04–8.35]
2009	7,416,605	517	6.97	7.38	[7.23–7.52]
2010	7,462,044	481	6.45	6.83	[6.7–6.98]
2011	7,501,853	543	7.24	7.64	[7.49–7.79]
2012	7,515,398	556	7.40	7.73	[7.58–7.88]
2013	7,478,968	616	8.24	8.57	[8.41–8.73]
2014	7,433,894	585	7.87	8.04	[7.89–8.2]
2015	7,424,754	683	9.20	9.23	[9.06–9.4]
2016	7,448,332	698	9.37	9.32	[9.16–9.49]
2017	7,496,276	618	8.24	8.08	[7.92–8.24]

Abbreviations: ASIR, age-standardised incidence rate.
